# Letrozole treatment of adult female mice results in a similar reproductive phenotype but distinct changes in metabolism and the gut microbiome compared to pubertal mice

**DOI:** 10.1186/s12866-019-1425-7

**Published:** 2019-03-12

**Authors:** Pedro J. Torres, Danalea V. Skarra, Bryan S. Ho, Lillian Sau, Arya R. Anvar, Scott T. Kelley, Varykina G. Thackray

**Affiliations:** 1 Department of Biology, San Diego State University, San Diego, CA USA; 2 Department of Obstetrics, Gynecology and Reproductive Sciences, University of California, San Diego, La Jolla, CA 92093 USA

**Keywords:** Gut microbiome, Polycystic ovary syndrome, Hyperandrogenism, Puberty

## Abstract

**Background:**

A majority of women with polycystic ovary syndrome (PCOS) have metabolic dysfunction that results in an increased risk of type 2 diabetes. We previously developed a pubertal mouse model using the aromatase inhibitor, letrozole, which recapitulates many of the reproductive and metabolic features of PCOS. To further our understanding of the effects of androgen excess, we compared the effects of letrozole treatment initiated in puberty versus adulthood on reproductive and metabolic phenotypes as well as on the gut microbiome.

**Results:**

Letrozole treatment of both pubertal and adult female mice resulted in reproductive hallmarks of PCOS, including hyperandrogenemia, anovulation and polycystic ovaries. However, unlike pubertal mice, treatment of adult female mice resulted in modest weight gain and abdominal adiposity, minimal elevation in fasting blood glucose and insulin levels, and no detectable insulin resistance. In addition, letrozole treatment of adult mice was associated with a distinct shift in gut microbial diversity compared to letrozole treatment of pubertal mice.

**Conclusions:**

Our results indicate that dysregulation of metabolism and the gut microbiome in PCOS may be influenced by the timing of androgen exposure. In addition, the minimal weight gain and lack of insulin resistance in adult female mice after letrozole treatment indicates that this model may be useful for investigating the effects of hyperandrogenemia on the hypothalamic-pituitary-gonadal axis and the periphery without the influence of substantial metabolic dysregulation.

**Electronic supplementary material:**

The online version of this article (10.1186/s12866-019-1425-7) contains supplementary material, which is available to authorized users.

## Background

Polycystic ovary syndrome (PCOS) is the most common endocrine disorder in reproductive-aged women with an estimated world-wide prevalence of 6–15%, but the etiology of PCOS is not well understood [1]. Heritability and twin studies have identified a strong genetic component that is likely polygenic [2–4]. Recent genome-wide association studies have reported multiple susceptibility loci associated with an increased risk of developing PCOS [5]. Environmental factors, such as prenatal exposure to androgens may also play a role in the etiology of PCOS [6]. Currently, diagnosis is made using the Rotterdam Consensus criteria (2003), which require at least two of the following: hyperandrogenism, oligo- or amenorrhea and polycystic ovaries [1].

Studies have shown that women with PCOS often suffer from profound, long-term health issues [7]. PCOS is the leading cause of anovulatory infertility in women and increases the likelihood of miscarriage and pregnancy complications [8, 9]. In addition, a majority of women with PCOS have abnormalities that increase their risk of developing metabolic disease [1, 10–15]. A large, retrospective study demonstrated that PCOS was associated with an increased risk of obesity (16 vs. 3.7%) and type 2 diabetes (12.5 vs. 3.8%) over a 15-year period [16]. Studies show that hyperandrogenism is strongly correlated with development of a metabolic phenotype. Metabolic dysfunction occurs predominantly in women diagnosed with hyperandrogenism and ovulatory dysfunction, independent of body mass index [17, 18].

A complex community of microorganisms (the microbiome) resides within the large intestine and is important for human health [19, 20]. Correlative studies have demonstrated that the gut microbiome of individuals with metabolic disorders, such as obesity and diabetes, differ significantly from healthy individuals [21–25]. In addition, mouse models of obesity are associated with gut microbiome dysregulation [26–31]. Studies have also shown that fecal transplantation of the gut microbiome from obese individuals into germ-free mice results in an obese phenotype [22, 32, 33], indicating a potential role of the gut microbiome in the development of metabolic disorders [34]. Recent studies indicate that changes in the gut microbiome are associated with PCOS. Women diagnosed with PCOS using the Rotterdam criteria were reported to have a significant reduction in the overall bacterial species richness (alpha diversity) of the gut microbial community and changes in the abundance of several bacterial taxa compared to healthy women [35–37]. Interestingly, a study from our lab also showed a significant correlation between hyperandrogenism and diversity of the gut microbiome, suggesting that androgens may influence the composition of the gut microbiome in women [37].

Since hyperandrogenism is associated with PCOS, researchers have created animal models to study the role of androgens in the development and pathology of PCOS [reviewed in [38–42]]. Several mouse models were developed using treatment with exogenous dihydrotestosterone but these models did not exhibit the elevated LH levels associated with PCOS [43–47]. We developed a PCOS mouse model in pubertal female mice using treatment with the aromatase inhibitor, letrozole, to limit the conversion of testosterone to estrogen which results in increased testosterone and decreased estrogen levels. This model is based on the findings that genetic variants of the aromatase gene are associated with the development of PCOS in women and that a higher androgen/estrogen ratio is found in the ovaries of women with PCOS [48–52]. We demonstrated that this mouse model has many hallmarks of PCOS including hyperandrogenemia, elevated LH levels, acyclicity, and polycystic ovaries [53, 54]. This model also exhibited a metabolic phenotype including weight gain, abdominal adiposity, dysglycemia, hyperinsulinemia, and insulin resistance after 5 weeks of letrozole treatment [55]. Similar to women with PCOS, we also showed that there was a significant decrease in the alpha diversity of the gut microbiome in the letrozole-induced PCOS mouse model that correlated with hyperandrogenism [54]. To gain more insight into the effects of androgen excess, we investigated whether the timing of testosterone exposure was important for the pathophysiology of PCOS by evaluating the effects of letrozole treatment on reproductive and metabolic phenotypes in pubertal versus adult female mice.

## Results

### Letrozole treatment of adult female mice resulted in reproductive hallmarks of PCOS

**Fig. 1:**
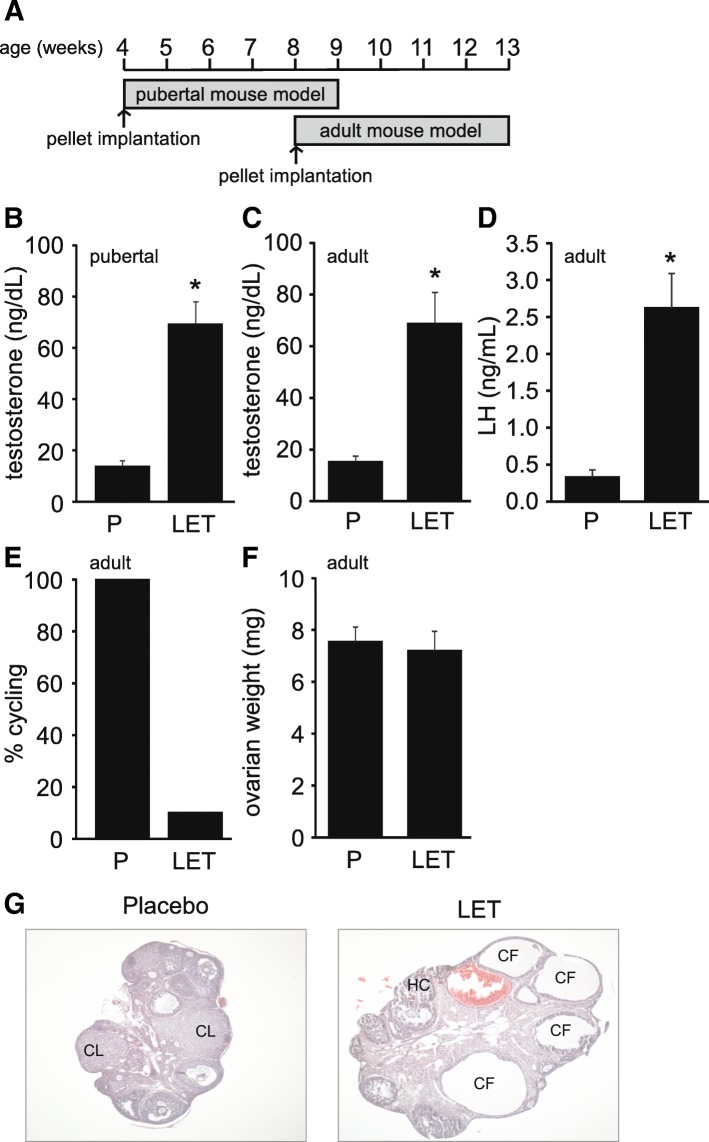
Letrozole treatment of adult female mice resulted in reproductive hallmarks of PCOS. Letrozole (LET) treatment was initiated at 8 weeks of age in the adult PCOS mouse model compared to 4 weeks of age in the pubertal PCOS mouse model (**a**). LET treatment of pubertal and adult female mice for 5 weeks resulted in elevated serum testosterone (**b-c**). LET treatment of adult female mice resulted in elevated LH levels (**d**), and decreased cyclicity as measured by percentage (%) of mice that had an estrous cycle between 4 and 5 weeks of treatment (**e**). In contrast to pubertal mice, LET treatment of adult female mice did not result in an increase in ovarian weight (**f**). Pubertal PCOS model (*n* = 24 placebo (P), *n* = 22 LET; adult PCOS model (*n* = 16 P, *n* = 14 LET). Student *t*-test; * *p* < 0.05. LET treatment of adult female mice resulted in ovaries lacking corpora lutea (CL) and containing cystic follicles (CF) and hemorrhagic cysts (HC) compared to placebo-treated mice (**g**)

In this study, we investigated whether the age at which letrozole treatment was initiated affected development of the PCOS phenotype in female mice (Fig. 1a). Five weeks of letrozole treatment in pubertal and adult female mice resulted in elevated serum testosterone levels (Fig. 1b-c). Letrozole treatment in adult female mice also resulted in increased LH levels (Fig. 1d) and acyclicity (Fig. 1e). Interestingly, the ovarian weight was similar in placebo and letrozole-treated adult mice (Fig. 1f). This is in contrast to the increase in ovarian weight previously observed in letrozole-treated pubertal mice [53, 54]. Similar to pubertal mice, letrozole treatment of adult female mice resulted in ovaries with cystic follicles and hemorrhagic cysts (Fig. 1g). Ovaries in the letrozole-treated mice also lacked corpora lutea, indicating a lack of ovulation compared to placebo-treated mice.

### Letrozole treatment of adult female mice resulted in minimal weight gain and abdominal adiposity after 5 weeks of treatment

**Fig. 2:**
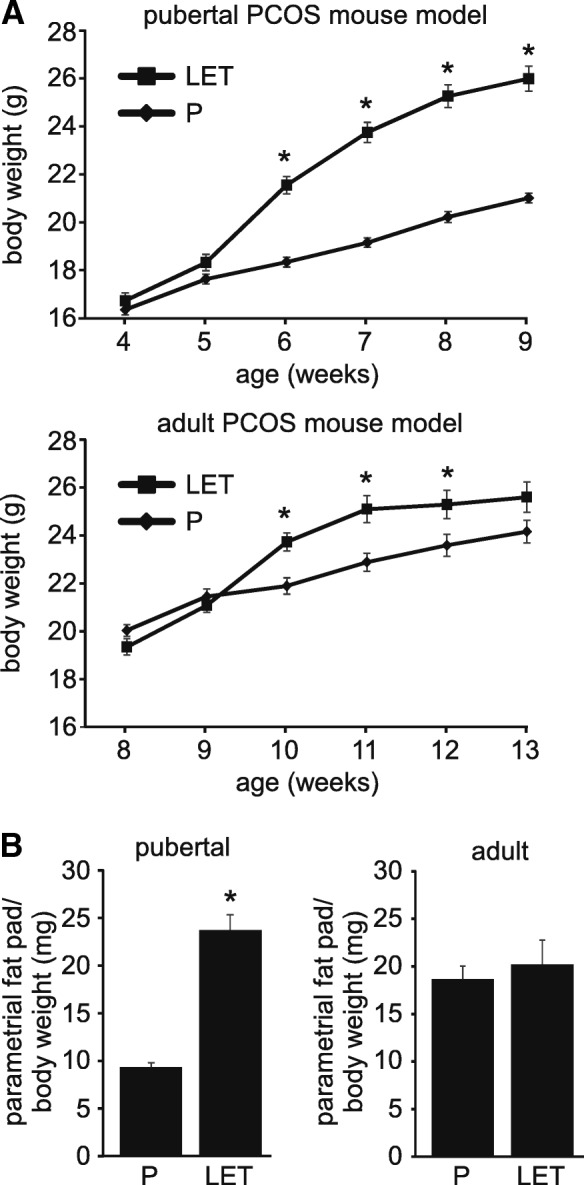
Five weeks of letrozole treatment of adult female mice did not result in substantial weight gain or abdominal adiposity. The phenotype of pubertal (4 week-old) versus adult (8 week-old) female mice treated with placebo (P) or letrozole (LET) for 5 weeks was compared. In contrast to pubertal mice, 5 weeks of LET treatment of adult female mice did not result in a significant increase in weight (**a**) or abdominal adiposity as measured by parametrial fat relative to total body weight (**b**). Pubertal PCOS model (n = 24 P, n = 22 LET; adult PCOS model (n = 16 P, n = 14 LET). Student *t*-test, * p < 0.05

Similar to previous reports [53, 54], letrozole treatment of pubertal female mice for 2 weeks resulted in substantial weight gain compared with placebo treatment, and weight was still increased at the end of the study (Fig. 2a). In contrast, letrozole treatment of adult female mice resulted in a more modest weight gain after 2 weeks of treatment and weight was not statistically different compared to placebo-treated mice after 5 weeks of treatment (Fig. 2a). Letrozole treatment of pubertal female mice resulted in a significant change in abdominal adiposity compared with placebo as reflected in an increase in the weight of the parametrial fat pad relative to total body weight (Fig. 2b). However, letrozole treatment of adult mice did not result in increased abdominal adiposity compared with placebo-treated mice (Fig. 2b).

### Letrozole treatment of adult female mice resulted in less elevation of fasting blood glucose and insulin levels and did not result in insulin resistance

**Fig. 3:**
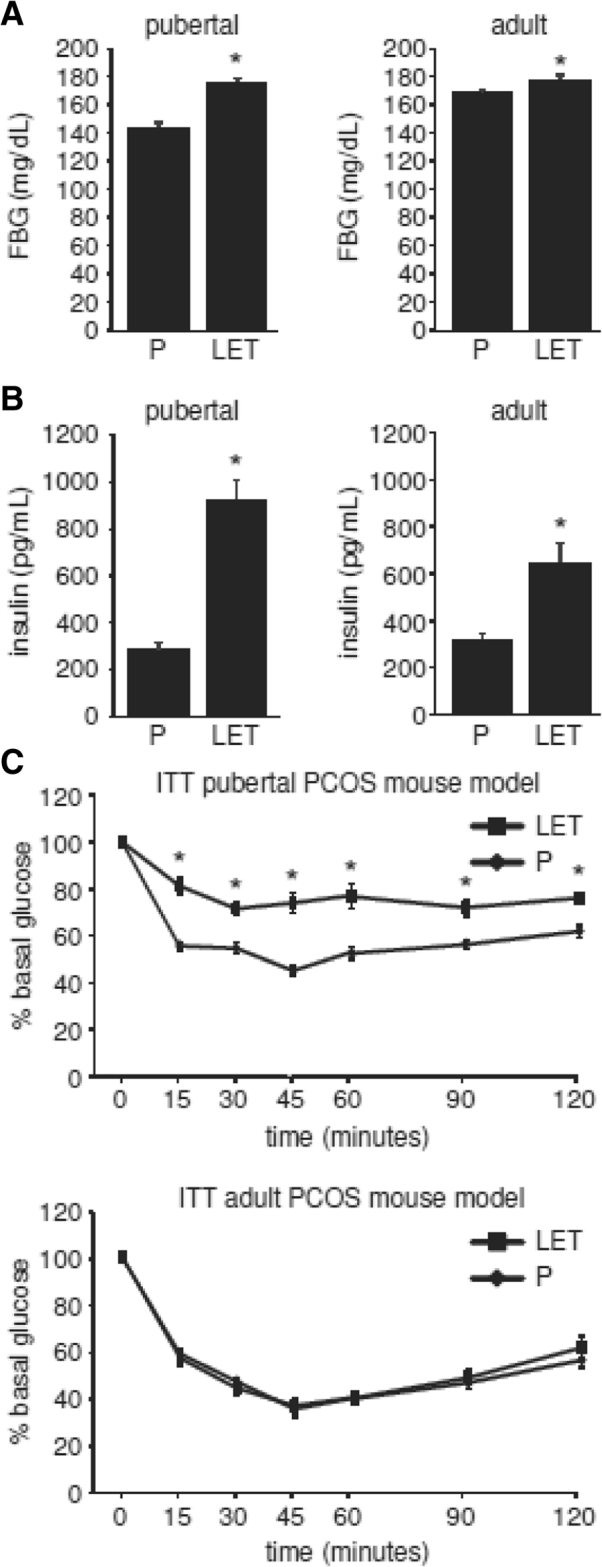
Five weeks of letrozole treatment of adult female mice resulted in a minimal increase in fasting blood glucose and insulin levels and did not result in insulin resistance. The metabolic phenotype of pubertal (4 week-old) versus adult (8 week-old) female mice treated with placebo (P) or letrozole (LET) for 5 weeks was compared. LET treatment of adult female mice resulted in reduced fasting blood glucose (FBG) or insulin levels compared to pubertal mice (**a-b**). Unlike pubertal female mice, LET treatment of adult female mice for five weeks did not result in insulin resistance (**c**). Pubertal PCOS model (*n* = 24 P, *n* = 22 LET; adult PCOS model (*n* = 8 P, *n* = 8 LET). Student *t*-test or two-way repeated-measures ANOVA with post-hoc Student *t*-tests to directly compare P versus LET at specific time points were performed; * *p* < 0.05

Both the pubertal and adult PCOS mouse models displayed dysglycemia and hyperinsulinemia but the phenotype was more modest in the adult model. Letrozole treatment of pubertal female mice resulted in elevated fasting blood glucose (FBG) levels and a 3-fold increase in fasting blood insulin levels (Fig. 3a-b). In contrast, letrozole treatment of adult female mice resulted in a slight but statistically significant increase in FBG and a 2-fold increase in insulin levels. There was no significant difference in the response to exogenous glucose in a glucose tolerance test in mice treated with letrozole compared to placebo in either the pubertal or adult PCOS mouse models (data not shown). Finally, the pubertal PCOS mouse model displayed signs of insulin resistance compared to placebo-treated mice while the adult PCOS mouse model remained insulin sensitive (Fig. 3c).

### Letrozole treatment of adult female mice was not associated with a strong correlation between alpha diversity and time

**Fig. 4:**
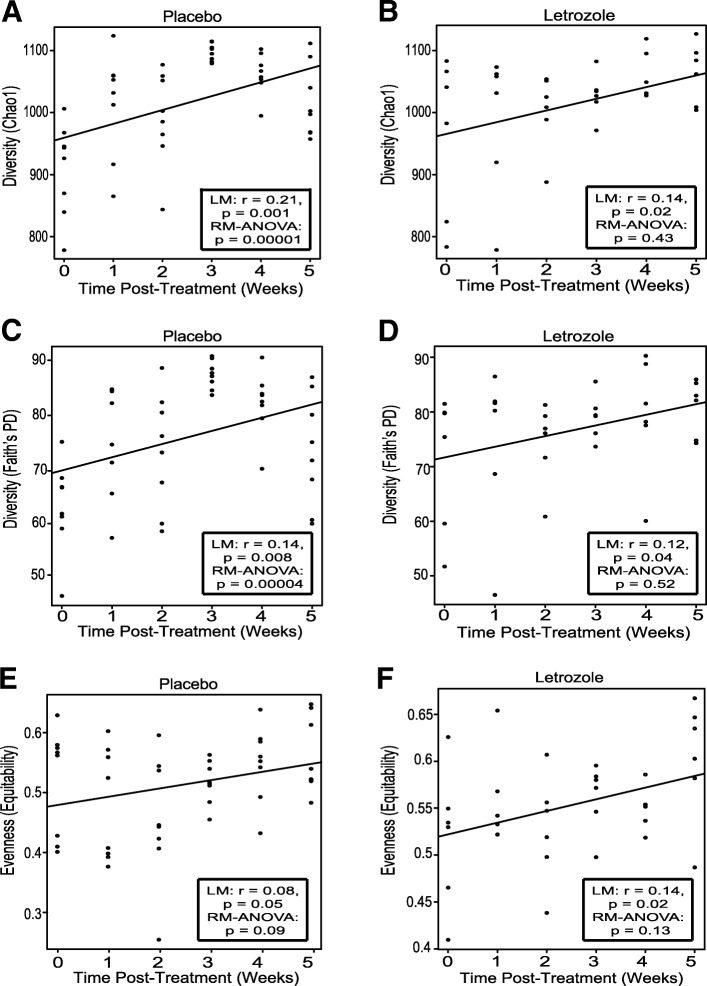
Letrozole treatment of adult female mice did not result in a strong correlation between time and alpha diversity of the gut microbial community. Chao 1 species richness estimate per sample at each collection time for placebo (**a**) and letrozole-treated adult female mice (**b**), (n = 8 placebo, *n* = 6 letrozole). Faith’s phylogenetic diversity (PD) estimate per sample at each collection time for placebo (**c**) and letrozole-treated adult female mice (**d**). Equitability (evenness) estimate per sample at each collection time point for placebo (**e**) and letrozole-treated mice (**f**). Line of best fit along with results of linear regression (LM) and repeated measures (RM) ANOVA are shown in box inset

Gut microbial diversity profiles were generated from 84 fecal samples taken prior to and during 5 weeks of placebo or letrozole treatment (weeks 0–5). Sequences collected before placebo and letrozole treatment were compared for both the pubertal and adult mouse models. No significant difference in alpha and beta diversity was observed between the two treatment groups at time 0, indicating that the gut microbiomes of the groups were similar prior to treatment for both the pubertal and adult cohort (Additional file 1: Figure S1). Similar to a previous study in pubertal mice [54], placebo-treated adult mice showed a strong positive correlation between alpha diversity and time as measured by species richness and phylogenetic diversity but not evenness of their gut communities (Fig. 4a, c, e). In contrast, letrozole treatment of adult mice was associated with a relatively weak positive correlation between alpha diversity and time (Fig. 3b, d, f). To examine this further, we evaluated whether there was a significant difference amongst the time points using a repeated measures (RM) ANOVA. RM-ANOVA found a highly significant effect of time on species richness and phylogenetic diversity in placebo-treated mice but no difference in letrozole-treated mice.

### Letrozole treatment of adult female mice resulted in changes in gut microbiome beta diversity

**Fig. 5:**
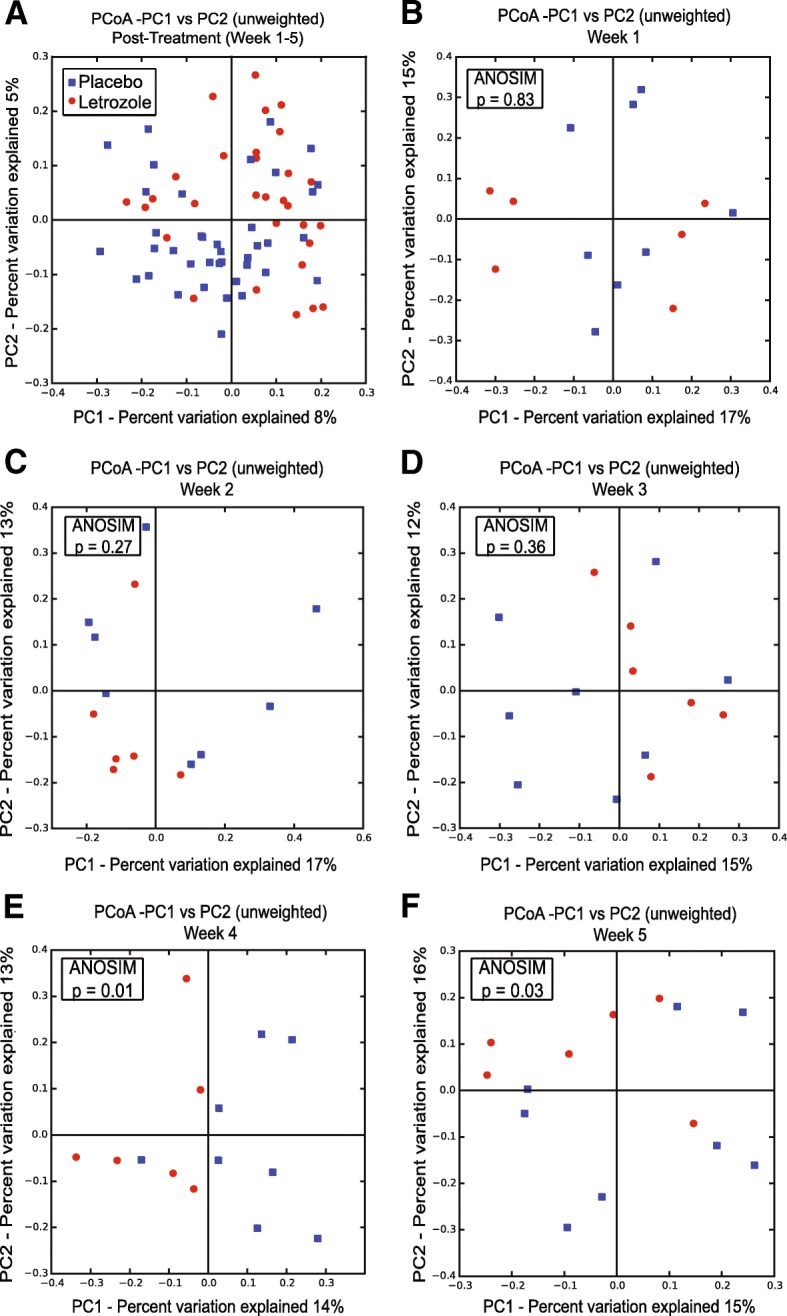
Letrozole treatment of adult female mice resulted in a significant shift in the beta diversity of the gut microbiome. Principal Coordinates Analysis (PCoA) of unweighted UniFrac for samples collected post-treatment (weeks 1–5) were compared between placebo (n = 8) and letrozole-treated (n = 6) mice (**a**). Proportion of variance explained by each principal coordinate axis is denoted in the corresponding axis label. Samples from placebo- and letrozole-treated mice were then compared for each time point (**b-f**). Results of Analysis of Similarity (ANOSIM) tests are shown in the box inset

UniFrac analyses were used to compare the similarity amongst gut microbial communities (beta diversity) in fecal samples from placebo versus letrozole–treated adult female mice. When all post-treatment data points were combined together, clustering of the data based on treatment was observed with unweighted UniFrac (Fig. 5a). When the samples were separated by the individual time points (Fig. 5b-f), Analysis of Similarity (ANOSIM) tests found a difference in the overall bacterial community composition of the gut microbiome between placebo and letrozole–treated adult female mice at weeks 4 and 5 post-treatment (*p* = 0.01 and *p* = 0.03 respectively). We also observed similar results using weighted UniFrac (data not shown).

### Distinct bacterial genera discriminated between placebo and letrozole treatment in the pubertal and adult PCOS mouse models

**Fig. 6:**
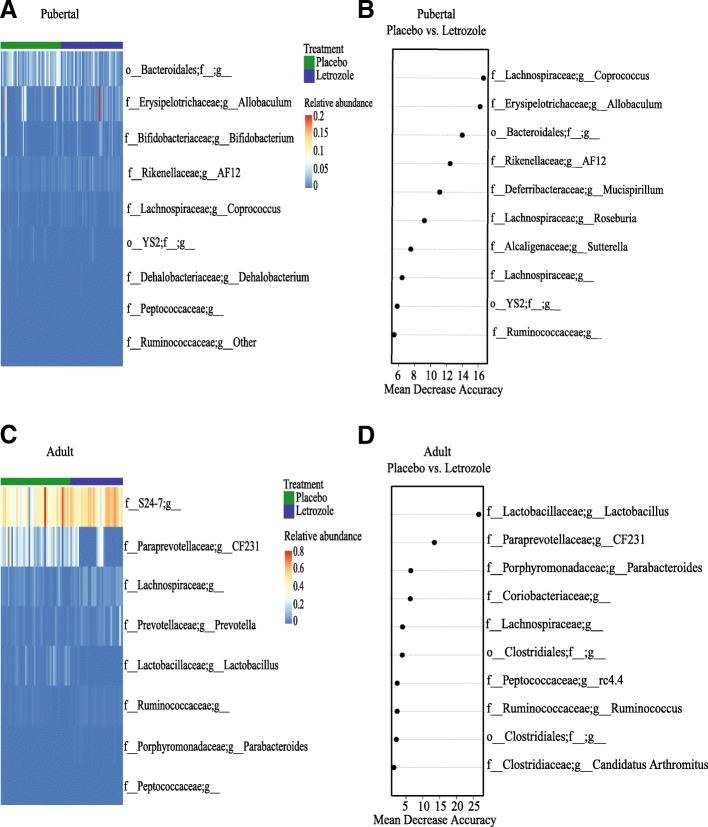
Repeated measures analysis of variance and Random Forest classification identified distinct bacteria associated with letrozole treatment in the pubertal versus adult PCOS mouse model. Repeated measures analysis of variance (corrected for multiple comparisons via FDR) was used to determine whether the abundance of specific bacterial genus differed in placebo versus letrozole-treated pubertal or adult mice. A heatmap was generated for the bacterial genera that had a FDR adjusted *p*-value of < 0.05 and mean taxa abundance above 0.001 (**a, c**). The Random Forest classifier was used to identify bacterial genera that distinguished between placebo and letrozole treatment in pubertal or adult female mice (**b, d**). The top ten most discriminant bacterial genera in the models were displayed in the variable importance plots using a total of forty-five genera in the analysis. An increase in mean decrease accuracy reflects the prediction strength of the variable in classifying the different treatment groups

In addition to studying changes in alpha and beta diversity, we also investigated whether the age at which letrozole treatment was initiated was important for changes in the taxonomic composition of the gut microbiome. We combined the post-treatment data (weeks 1–5) from placebo and letrozole-treated mice in the pubertal and adult mouse models. Based on the Greengenes taxonomic database, we identified a total of 10 bacterial phyla and 51 bacterial genera in the four different groups. Similar to our previous study [54], the majority of Operational Taxonomic Units (OTUs) in the adult mouse fecal samples were identified as Bacteriodetes or Firmicutes (~ 84–95%). We used RM-ANOVA to determine if the mean relative abundances of specific bacterial genera were different in the gut microbiome of placebo versus letrozole-treated mice in the pubertal and adult mouse models. A heatmap was generated to represent the relative abundance of 9 different bacterial genera that changed significantly with letrozole treatment (FDR-corrected *p* < 0.05) in the pubertal mouse model (Fig. 6a). Letrozole treatment of pubertal female mice resulted in higher relative abundances of *Coprococcus, Allobaculum, Bifidobacterium,* and an undescribed genus belonging to the Ruminococcaceae, as well as a lower abundance of *AF12*, *Dehalobacterium,* taxa belonging to the uncultured order YS2, and undescribed genera of Peptococcaceae and Bacteroidales (Fig. 6a).

In contrast to the pubertal mice, letrozole treatment of adult female mice resulted in changes in the mean relative abundance of a distinct set of 8 bacterial genera (FDR-corrected p < 0.05). With the exception of uncultured members of the genus-level *CF231* group*,* the rest of the genera from the Bacteroidetes phylum increased with letrozole treatment in adult female mice, including *Prevotella,* an uncultured genus within Parabacteroides and a genus-level group within the S24–7 family (Fig. 6c). Letrozole treatment of adult mice also resulted in a higher relative abundance of genera from Lachnospiraceae, Ruminococcaceae, and Peptococcaceae, as well as a lower abundance of *Lactobacillus* (Fig. 6c).

### Random Forest classifier identified bacterial genera predictive of placebo and letrozole treatment in the pubertal and adult PCOS mouse models

The Random Forest (RF) classifier was trained to determine how well placebo or letrozole treatment could be predicted based on bacterial relative abundances in the two models (pubertal and adult). Forty-five of the 51 total bacterial genera identified in the four different groups were used for RF classification (six were excluded due to low relative abundances). Our results showed that RF predicted treatment category in the pubertal group with 78.5% accuracy while it predicted treatment category in the adult group with 84% accuracy (Table 1). Variable importance by mean decrease in accuracy was calculated for the RF models. Figure 6b and d illustrate 10 bacterial genera whose removal caused the greatest decrease in model accuracy (i.e. the most important for classification) in the pubertal and adult mouse models respectively. In the pubertal model, the removal of *Coprococcus*, *Allobaculum*, *AF12*, *Mucispirillum*, *Roseburia*, *Sutterella*, and an unknown genus from Bacteroidales had the greatest impact on classification (mean decrease accuracy > 8; Fig. 6b). In the adult mice, the removal of *Lactobacillus*, *CF231* and *Parabacteriodes* caused the greatest decrease in prediction accuracy (mean decrease accuracy > 8; Fig. 6d)*.*

**Table 1:** Classification error rates carried out using Random Forest classifiers composed of 500 trees

	Predicted classes		Classification error rates	OOB estimate of error rate	Accuracy
	Placebo	Letrozole			
Pubertal				21.5%	78.5%
Placebo	32	7	0.18		
Letrozole	10	30	0.25		
Adult				16.0%	84.0%
Placebo	30	1	0.03		
Letrozole	7	13	0.35		

## Discussion

Our study demonstrated that initiation of letrozole treatment during puberty or adulthood in female mice resulted in reproductive hallmarks of PCOS, including elevated testosterone levels, anovulation and ovaries with cystic follicles. This suggests that the timing of androgen exposure (puberty versus adulthood) may not be important for development of the PCOS reproductive phenotype. On the other hand, our study did find a clear divergence between the metabolic phenotypes of the pubertal and adult mouse models. Similar to previously published studies [53, 54], letrozole treatment in pubertal female mice resulted in multiple metabolic features of PCOS, including obesity, abdominal adiposity, hyperinsulinemia, and insulin resistance. On the other hand, letrozole treatment in adult female mice did not result in substantial weight gain, abdominal adiposity or insulin resistance, indicating that androgen exposure is not sufficient to induce the full PCOS-like metabolic phenotype in adult female mice. Interestingly, studies using post-natal treatment with DHT to create a hyperandrogenic mouse model observed a similar pattern: the metabolic phenotype depended on when DHT treatment was initiated. Compared with placebo-treated mice, female mice treated with DHT starting at 3 weeks of age gained significantly more weight, had greater levels of abdominal adiposity and were glucose intolerant [43, 47]. In contrast, while female mice treated with DHT in adulthood had impaired glucose tolerance, they did not become obese or display increased abdominal adiposity [56].

Our results also suggest that the timing of excess androgen exposure may be an important component in the development of the PCOS metabolic phenotype. Since PCOS often manifests in the early reproductive years, puberty has been suggested to be a critical developmental time period for the development and pathology of PCOS [6, 57]. Indeed, PCOS has been hypothesized to originate from abnormal pubertal development due to a lack of transition from an androgen-dominated state in early puberty to an estrogenic state in late puberty [58, 59]. Puberty is a time of considerable hormonal and metabolic change, including an increase in insulin resistance [60]. Although physiological insulin resistance is common in healthy adolescents, it usually resolves to prepubertal levels in adulthood [61]. Pubertal insulin resistance has been reported to increase the risk of developing type 2 diabetes along with accelerating the complications of diabetes [62–65]. Thus, it is possible that insulin resistance and the hyperinsulinemia that occurs during puberty may also contribute to the risk of developing obesity and metabolic dysfunction in PCOS.

Another factor that changes during the transition from childhood to adulthood is the gut microbiome. Studies have shown that children or adolescents have a distinct gut microbial community compared to adults [66, 67]. Moreover, prepubertal mice were reported to have a different gut microbiome than adult mice [68, 69]. Studies in humans and mice have shown a strong positive association between gut bacterial alpha diversity and age, indicating that the complexity of the gut microbiome increases as the host ages [70–72]. In contrast to placebo, there was no significant effect of time on alpha diversity in letrozole-treated mice when the data was adjusted for within subject error using RM-ANOVA (Fig. 4) [54]. With regards to beta diversity, letrozole treatment of both pubertal and adult female mice resulted in a distinct shift in the gut microbial composition (Fig. 5). However, closer examination of the types of bacteria that changed after letrozole treatment showed that the taxa driving the shift in beta diversity were quite distinct in the two mouse models (Fig. 6).

Letrozole treatment initiated during puberty resulted in changes in the abundances of bacterial genera previously reported to be altered in diet-induced obesity mouse models. In the pubertal model, RF and statistical analysis of relative bacterial abundances determined that *Coprococcus, Allobaculum* and an unknown genus from Bacteroidales differentiated the gut microbiomes of placebo and letrozole-treated mice (Fig. 6a and b). Significant differences were also observed in the relative abundance of *Bifidobacterium*, reported to have strain-specific effects on weight gain in rodents [73], as well as *Dehalobacterium* and unknown genera belonging to the Rikenellaceae and Ruminococcaceae families, all of which have been associated with obesity [28, 74–77]. The genus with the strongest effect on RF classification, namely *Coprococcus*, was previously reported to be more abundant in obese individuals [78, 79], in agreement with the higher levels observed after letrozole treatment in pubertal mice. The second most important genus in terms of classification, *Allobaculum*, was reported to be lower in the gut of obese mice fed a high-fat diet [80, 81], in contrast to the increase in *Allobaculum* observed after letrozole treatment.

In comparison to pubertal mice, letrozole treatment of adult female mice had a distinct impact on the composition of the gut microbial community. With the exception of a genus within the Peptococcaceae, the bacterial genera most affected by letrozole treatment in adult females were not altered in pubertal mice and vice versa (Fig. 6a, c). It should be noted that the genera that changed in the pubertal and adult female mice after letrozole treatment were present at both ages, indicating that the differential effects of letrozole treatment in the two models was not due to the absence of specific bacteria. The most striking difference in letrozole treatment of adult female mice was the importance that *Lactobacillus, Parabacteroides* and the uncultured Paraprevotellaceae group *CF231* played in classifying the treatment groups (Fig. 6d). The mean relative abundance of these bacteria changed significantly after letrozole treatment in adult female mice (Fig. 6c). This is in contrast to the increased abundance of some *Lactobacillus* species observed in obese humans [82–84], though direct comparisons are difficult since there may be strain-specific effects of *Lactobacillus* on weight gain [85]. While *CF231* has not been described in much detail, members of the Paraprevotellaceae are found in the gut of many mammals [86, 87] and have been suggested to be involved in the degradation of plant polysaccharides into short chain fatty acids [88]. *Parabacteroides* are also known to metabolize non-digestible carbohydrates, but the increase in *Parabacteroides* relative abundance after 5 weeks of letrozole treatment contrasts with the decrease observed in mice fed a high-fat diet [27, 89].

## Conclusions

In summary, our study demonstrated that the timing of androgen exposure may be important for development of the PCOS metabolic phenotype and associated changes in the gut microbiome. While letrozole treatment of female mice during puberty and in adulthood both resulted in reproductive hallmarks of PCOS, including hyperandrogenemia, anovulation and polycystic ovaries, letrozole treatment in adulthood did not result in the weight gain, abdominal adiposity or insulin resistance observed in the pubertal PCOS mouse model. In addition, letrozole treatment in adulthood resulted in distinct changes in the gut microbiome, particularly in *Lactobacillus*. Although evidence is accumulating that changes in steroid hormones are associated with an altered gut microbiome [90], the mechanisms involved in steroid hormone/gut microbe interactions are currently unknown. Future studies investigating whether steroid hormones regulate the gut microbiome through actions in the gastrointestinal tract, immune system or other tissues will begin to address the mechanisms involved.

Given that many of the previous studies that report an association of specific bacterial genera with obesity in humans and high fat diet-induced mouse models are contradictory, it is possible that these results are due to modulation of specific bacterial species and strains within genera. Future studies should employ higher resolution methods such as metagenomic sequencing or quantitative PCR to fully understand the effects of hyperandrogenism on the gut microbiome. Moreover, since many studies of the role of the gut microbiome in obesity are confounded by the effect of diet on the microbiome, the letrozole-induced PCOS mouse model provides an opportunity to study the effects of androgen excess on the gut microbiome and metabolism in a diet-independent setting, since food intake is not altered by letrozole treatment [55]. Moreover, the adult PCOS mouse model can be used to study the effects of hyperandrogenism in female mice without the confounding variable of insulin resistance. Further studies addressing whether the gut microbiome plays a causal role in the development of PCOS or if manipulation of the gut microbiome can improve the PCOS phenotype will be informative. In addition, prospective studies with adolescent girls may be crucial to understand the etiology and development of PCOS, particularly the metabolic dysregulation and changes in the gut microbiome associated with this disease.

## Methods

### PCOS mouse model

C57BL/6NHsd female mice purchased from Envigo were housed in a vivarium for one week under specific pathogen-free conditions with an automatic *12 h*:*12 h light*/*dark* cycle (*light* period: 06.00–18.00) and ad libitum access to water and food (Teklad Global 18% Protein Extruded Diet, Envigo). Prior to the beginning of the study, the mice were sorted by weight to ensure that the starting weight was similar between the two treatment groups. To establish the pubertal or adult PCOS models, 4 or 8 week-old female mice, respectively were implanted subcutaneously with a placebo or 3 mg letrozole pellet (3 mm diameter; Innovative Research of America) that provided a slow, constant release of letrozole (50 μg/day) over 5 weeks. For the duration of the experiment, the mice were housed 2 per cage: 2 placebo or 2 letrozole-treated mice. Placebo and letrozole-treated mice were not housed together to avoid the influence of coprophagy on the PCOS mouse model. At the end of the study, the mice were sacrificed using 2.5% isoflurane delivered with a precision vaporizer followed by a physical method of euthanasia.

### Analysis of reproductive and metabolic phenotype

The mice were weighed weekly. The stage of the estrous cycle for placebo and letrozole-treated mice was determined from the predominant cell type in vaginal epithelium smears obtained during weeks 4–5 of treatment. After 5 weeks of placebo or letrozole treatment, the mice were fasted for 5 h and blood from the tail vein was collected to measure fasting insulin levels. Blood glucose was measured using a handheld glucometer (One Touch UltraMini, LifeScan, Inc) and an intraperitoneal (IP) insulin tolerance test (ITT) was performed. Tail vein blood glucose was measured just before (time 0) an IP injection of insulin (0.75 U/kg in sterile saline; Humulin R U-100, Eli Lilly) was given and at 15, 30, 45, 60, 90, and 120 min post injection.

At the end of the experiment, blood was collected from the posterior vena cava, parametrial fat pads were dissected and weighed, and the ovaries were dissected, weighed, fixed in 4% paraformaldehyde at 4 °C overnight, and stored in 70% ethanol before processing for histology. Paraffin-embedded ovaries were sectioned at 10 μm and stained with hematoxylin and eosin (Zyagen). Serum testosterone were measured using a mouse ELISA (range 10–800 ng/dL) while LH levels were measured using a radioimmunoassay (range 0.04–75 ng/mL) by the University of Virginia Center for Research in Reproduction Ligand Assay and Analysis Core Facility. Serum insulin was measured using a mouse ELISA (ALPO) by the University of California, Davis Mouse Metabolic Phenotyping Center. The data from four mice in the pubertal and adult letrozole-treated groups were removed from the analyses because these mice did not have a significant elevation in serum testosterone when compared to the average of the placebo-treated mice. The analysis of the reproductive and metabolic phenotypes was performed with data from 2 unpublished cohorts of the adult PCOS model (total *n* = 16 placebo, *n* = 14 letrozole) and 3 cohorts of the pubertal PCOS model (total *n* = 24 placebo, *n* = 22 letrozole) including 2 unpublished and 1 previously published cohort [54]. Differences between placebo and letrozole treatment were analyzed by Student *t*-test or two-way repeated measures ANOVA followed by post-hoc comparisons of individual time points.

### Fecal sample collection and DNA isolation

Fecal samples were collected from one cohort of 8-week-old female mice (*n* = 8/group) prior to treatment with placebo or letrozole and once per week thereafter for 5 weeks. Fecal samples were frozen immediately after collection and stored at − 80 °C. Bacterial DNA was extracted from the fecal samples using the MoBio PowerSoil DNA Extraction Kit following the manufacturer’s protocol, and the DNA was stored at − 80 °C.

### 16S rRNA amplicon sequencing

The V4 hypervariable region of the 16S rRNA gene was PCR amplified with primers 515F (GTGCCAGCMGCCGCGGTAA) and 806R (GGACTACHVGGGTWTCTAAT) [91]. The reverse primers contained unique 12 base pair Golay barcodes that were incorporated into the PCR amplicons [92]. The barcoded primers allowed for pooling of multiple PCR amplicons in a single sequencing run. Thermocycling parameters were as follows: denaturing at 98 °C for 2 min followed by amplification for 35 cycles at 98 °C for 30 s, 50 °C for 30 s and 72 °C for 60 s, and a final extension of 72 °C for 10 min. The resulting amplicons were submitted to The Scripps Research Institute Next Generation Sequencing Core Facility where they were cleaned using Zymo DNA Clean & Concentrator™-25 columns, quantified using a Qubit Flourometer (Life Technologies) and pooled. Pooled PCR products were size selected on a 2% agarose gel (290–350 bp), purified using a Zymoclean™ Gel DNA recovery kit and used to prepare sequencing libraries following the recommended Illumina protocol involving end-repair, A-tailing and adapter ligation. The DNA library was then size selected on a 2% agarose gel (410–470 bp), cleaned using the Agencourt SPRI system (Beckman Coulter, Inc.) and PCR amplified with HiFi Polymerase (Kapa Biosystems) for 12 cycles. The amplified DNA products were again size selected on a 2% agarose gel and purified using the Zymoclean™ Gel DNA recovery kit. The purified DNA library was quantitated, denatured in 0.1 N NaOH and diluted to a final concentration of 5 pM before being loaded onto the Illumina single read flow-cell for sequencing on the Illumina MiSeq system along with 4 pM PhiX control library.

### 16S rRNA gene sequence analysis

16S rRNA sequences for the adult mice were de-multiplexed using the Quantitative Insights Into Microbial Ecology (QIIME v.1.9.1, http://www.qiime.org) pipeline [93] using the default split_libraries.py script parameter [94]. This resulted in approximately 4.3 million Illumina sequences across all samples with an average of 50,000 sequences per sample. Sequences from two mice in the letrozole-treated group were removed from the analysis because these mice did not have a significant elevation in serum testosterone levels compared to the average of the placebo-treated mice. The 16S rRNA gene sequencing quality control and analysis for the samples from the adult mice followed the same pipeline as the samples in a previously published study with placebo or letrozole-treated pubertal female mice [54]. Sequences were clustered using the pick_de_novo_otus.py script with usearch [95]. Sequences were assigned to OTUs with an assumed 97% threshold of pairwise identity for bacterial species by comparison with the Greengenes reference database [96] using the RDP classifier [97]. Before performing downstream analysis, singletons and OTUs present in less than 25% of the samples were discarded from the database to minimize the effect of spurious, low abundance sequences using the filter_otus_from_otu_table.py script. Sequences were then aligned using PyNast [93] and a phylogenetic tree constructed using FastTree [98]. The alpha_diversity.py script was used to estimate several different attributes of alpha diversity. Species richness was estimated using Chao1 to define the total number of unique species in a community [99]. Faith’s Phylogenetic Diversity was used to measure the phylogenetic diversity of a community by calculating the total branch lengths on a phylogenetic tree of all members of the community [100]. Evenness was estimated using the Equitability index [101]. The beta_diversity_through_plots.py script was used to compute weighted and unweighted UniFrac distances [102]. The smaller the UniFrac distance between two microbial communities, the more similar the communities are in their overall diversity. The weighted UniFrac distance metric incorporates the abundance of specific taxa in each community into the UniFrac distance calculation while unweighted UniFrac ignores abundance information. Taxonomic distributions across sample categories were calculated (from phylum to genus) using the summarize_taxa_through_plots.py script. Several bacterial genera such as *Anaeroplasma* and an unknown Enterobacteriaceae were excluded from the analysis because of extremely low abundance, suggesting that they may have been artifacts. Sequences from placebo-treated samples collected during week 5 (9 weeks of age) of the pubertal cohort were compared to samples collected from week 1 (9 weeks of age) from the adult cohort. No significant difference in alpha and beta diversity was observed between the two cohorts, indicating that the gut microbiome at the end of placebo treatment in the pubertal cohort was similar to the gut microbiome at the beginning of placebo treatment in the adult cohort.

### Statistical analysis

Pearson’s product-moment correlation was performed when analyzing alpha diversity over time using the RStudio statistical package (version 0.99.893). RM-ANOVA was used to model alpha diversity measures accounting for within subject error. Two-dimensional PCoA plots were constructed using the make_2d_plots.py script in QIIME. ANOSIM tests for weighted and unweighted UniFrac distances between treatments were performed using the compare_categories.py script. The biom table of post treatment samples (weeks 1–5) from the adult study was merged with the biom table from the pubertal study, resulting in approximately 6.2 million sequences from 170 samples (pubertal = 100 samples; adult = 70 samples). The merged dataset was used to compare differences among treatment group and developmental stage. RM-ANOVA adjusting for within subject error (corrected for multiple comparisons via FDR) was used to determine whether the abundance of specific bacterial genus differed between treatments. The RF supervised machine-learning classifier was used to determine how well a given set of factors (e.g. bacterial genera) classified discrete categories and which factors were most important for the classification [70, 116]. RF was implemented in R using the “randomForest” library, and was used to identify bacterial genera that differentiated placebo and letrozole treatment within pubertal or adult mice.

## Additional file

Additional file 1: **Figure S1.** No differences in gut microbial community diversity between placebo and letrozole-treated mice were observed prior to treatment. No significant differences in gut microbiome alpha diversity (Faith’s PD) between placebo- and letrozole-treated mice were observed prior to treatment (week 0) in the pubertal (placebo *n* = 10, letrozole n = 10) (A) or adult (placebo *n* = 8, letrozole *n* = 6) (B) model. Similarly, no differences in beta diversity (unweighted UniFrac) were observed between placebo- and letrozole-treated mice prior to treatment in the pubertal (C) or adult model (D). Student *t*-test was used to compare alpha diversity between groups and Analysis of Similarity (ANOSIM) test was used to compare beta diversity between groups. (PDF 1335 kb)

## Abbreviations

ANOSIM: Analysis of similarity
FBG: Fasting blood glucose
IP: Intraperitoneal
ITT: Insulin tolerance test
OTU: Operational taxonomic unit
PCOS: Polycystic ovary syndrome
QIIME: Quantitative Insights Into Microbial Ecology
RF: Random Forest
RM-ANOVA: Repeated measures analysis of variance
