# Supplementary figures and images for: Letrozole treatment of adult female mice results in a similar reproductive phenotype but distinct changes in metabolism and the gut microbiome compared to pubertal mice
Source: BMC Microbiol. 2019 Mar 12;19:57. doi: 10.1186/s12866-019-1425-7 (PMC6419356; doi:10.1186/s12866-019-1425-7)

FigureS1

A

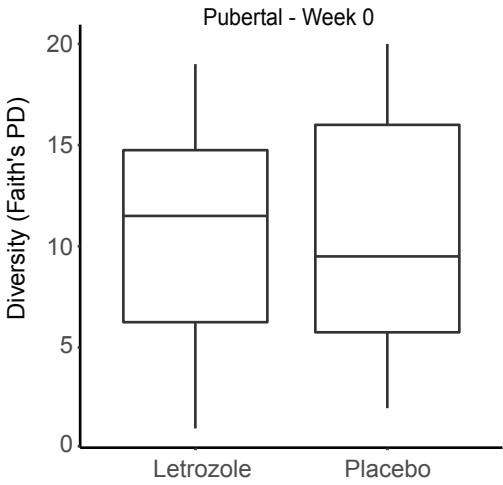

B

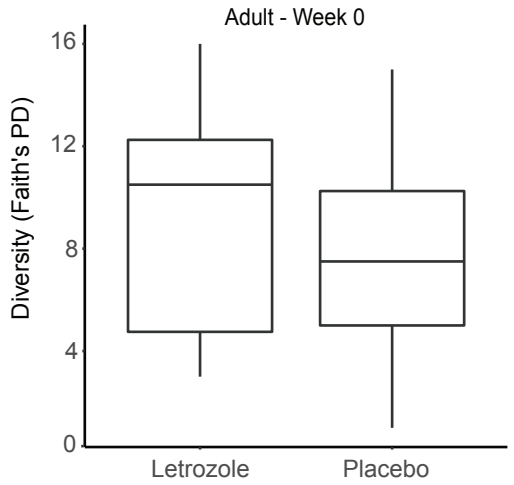

C

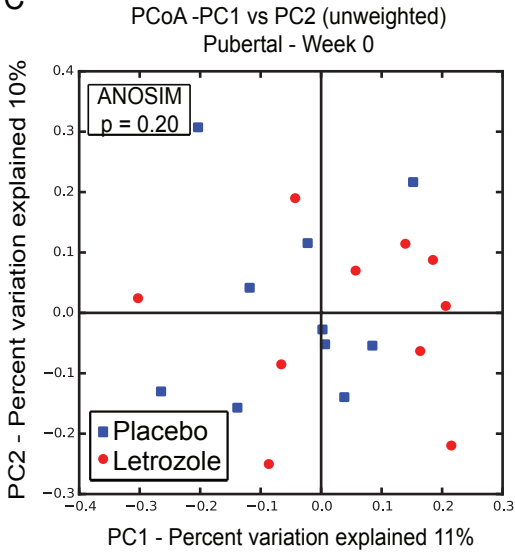

D

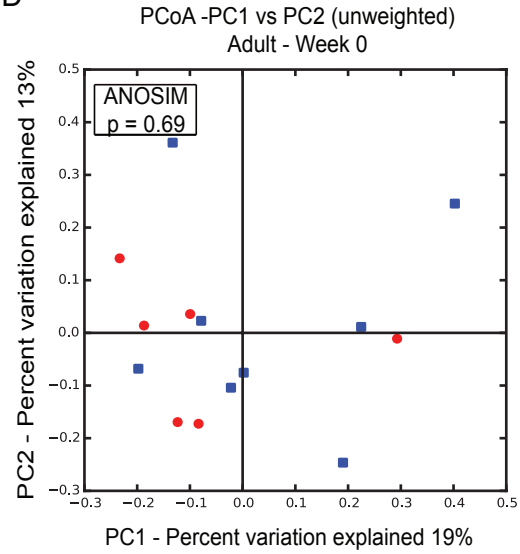

Supplement: Supplementary file 1 — Figure S1. No differences in gut microbial community diversity between placebo and letrozole-treated mice were observed prior to treatment. No significant differences in gut microbiome alpha diversity (Faith’s PD) between placebo- and letrozole-treated mice were observed prior to treatment (week 0) in the pubertal (placebo n = 10, letrozole n = 10) (A) or adult (placebo n = 8, letrozole n = 6) (B) model. Similarly, no differences in beta diversity (unweighted UniFrac) were observed between placebo- and letrozole-treated mice prior to treatment in the pubertal (C) or adult model (D). Student t-test was used to compare alpha diversity between groups and Analysis of Similarity (ANOSIM) test was used to compare beta diversity between groups. (PDF 1335 kb) [file 12866_2019_1425_MOESM1_ESM.pdf]
